# Cortical Thickness Related to Compensatory Viewing Strategies in Patients With Macular Degeneration

**DOI:** 10.3389/fnins.2021.718737

**Published:** 2021-10-01

**Authors:** Tina Plank, Edith M. A. Benkowitsch, Anton L. Beer, Sabine Brandl, Maka Malania, Sebastian M. Frank, Herbert Jägle, Mark W. Greenlee

**Affiliations:** ^1^Institute of Experimental Psychology, University of Regensburg, Regensburg, Germany; ^2^Department of Ophthalmology, University Hospital Regensburg, Regensburg, Germany; ^3^Department of Psychological and Brain Sciences, Dartmouth College, Hanover, NH, United States; ^4^Department of Cognitive, Linguistic & Psychological Sciences, Brown University, Providence, RI, United States

**Keywords:** macular degeneration, central vision loss, cortical thickness (CT), visual cortex, cortical eye fields, magnetic resonance imaging (MRI)

## Abstract

Retinal diseases like age-related macular degeneration (AMD) or hereditary juvenile macular dystrophies (JMD) lead to a loss of central vision. Many patients compensate for this loss with a pseudo fovea in the intact peripheral retina, the so-called “preferred retinal locus” (PRL). How extensive eccentric viewing associated with central vision loss (CVL) affects brain structures responsible for visual perception and visually guided eye movements remains unknown. CVL results in a reduction of cortical gray matter in the “lesion projection zone” (LPZ) in early visual cortex, but the thickness of primary visual cortex appears to be largely preserved for eccentric-field representations. Here we explore how eccentric viewing strategies are related to cortical thickness (CT) measures in early visual cortex and in brain areas involved in the control of eye movements (frontal eye fields, FEF, supplementary eye fields, SEF, and premotor eye fields, PEF). We determined the projection zones (regions of interest, ROIs) of the PRL and of an equally peripheral area in the opposite hemifield (OppPRL) in early visual cortex (V1 and V2) in 32 patients with MD and 32 age-matched controls (19–84 years) by functional magnetic resonance imaging. Subsequently, we calculated the CT in these ROIs and compared it between PRL and OppPRL as well as between groups. Additionally, we examined the CT of FEF, SEF, and PEF and correlated it with behavioral measures like reading speed and eccentric fixation stability at the PRL. We found a significant difference between PRL and OppPRL projection zones in V1 with increased CT at the PRL, that was more pronounced in the patients, but also visible in the controls. Although the mean CT of the eye fields did not differ significantly between patients and controls, we found a trend to a positive correlation between CT in the right FEF and SEF and fixation stability in the whole patient group and between CT in the right PEF and reading speed in the JMD subgroup. The results indicate a possible association between the compensatory strategies used by patients with CVL and structural brain properties in early visual cortex and cortical eye fields.

## Introduction

In Western countries, macular degeneration is one of the most common causes for (partial) blindness, especially in elderly people (Ambati and Fowler, 2012). Thereby, the most important part of the retina – the macula – degenerates due to regional atrophy (Kellner et al., 2004). The visual deficit in age-related macular degeneration (AMD) is characterized by atrophy of photoreceptor cells in the patients’ macula resulting in a complete foveal scotoma. Also some hereditary, juvenile forms of retinal dystrophies (JMD, e.g., Stargardt’s disease or cone-rod dystrophy) lead to central scotomas due to similar processes (Glazer and Dryja, 2002). Degenerative diseases, such as macular degeneration, physiologically lead to a structural and functional change in the associated areas of the brain (e.g., Xiao et al., 2007; Xie et al., 2007; Barnes et al., 2010; Hernowo et al., 2014). The study of changes in the thickness of cortical gray matter can serve as an indication of neural plasticity as a consequence of central vision loss. Since previous findings at the neuronal level indicate that despite disease, signals from the retina can still be transmitted to the visual cortex for subsequent processing, further investigation into the capacity of neural plasticity will inform future therapy methods and visual restoration (Prins et al., 2016a; McGregor et al., 2020).

Although macular degeneration is primarily a retinal disease, reductions of the gray matter volume (Plank et al., 2011; Hernowo et al., 2014) and density (Boucard et al., 2009) as well as cortical thinning (Prins et al., 2016b) in the lesion projection zone (LPZ) of the primary (V1) and secondary (V2) visual cortex were observed in both AMD and JMD patients. For example, Plank et al. (2011) investigated the structural changes of the brain as a consequence of sensory deprivation as it occurs in hereditary retinal dystrophies (JMD) with central visual loss (e.g., in Stargardt’s disease or cone-rod dystrophy). Since a large part of the responsible cortex is not sufficiently stimulated as a consequence of the loss in central visual field processing, there is a decrease of gray matter in these regions. Particularly in the occipital pole region along the posterior calcarine sulcus, the patient group showed a significant reduction in gray matter volume. This region corresponds to the foveal representation zone (or lesion projection zone, LPZ), which comes about as the consequence of macular degeneration as a lesion of the fovea. Similar results were found by Prins et al. (2016b), who studied the cortical thickness (CT) in visual cortex (V1 and V2) in AMD and JMD patients as well as in healthy controls. They found a reduction of CT in the patient group, that was most pronounced in the posterior parts of V1 and V2. Burge et al. (2016), who compared 10 MD-patients to age-, gender- and education-matched healthy controls, reported that CT in patients decreased in the area of central vision representation, but increased in peripherally responsive visual cortex areas compared to the controls. Furthermore, in studies of other diseases with visual loss, such as primary open-angle glaucoma, changes of the CT in the associated areas were also detected as a result of the impairment (Yu et al., 2013). Thus, the examination of CT in patients with central vision loss is of great importance to understand the potential of neural plasticity in visual restoration.

Patients with central vision loss are forced to develop specific coping strategies. Many patients compensate for impaired central vision by using strategies of eccentric viewing to manage daily visual tasks such as reading. They often develop a pseudo fovea at a specific area of their intact peripheral retina, the so-called “preferred retinal locus” (PRL; e.g., Bäckman and Inde, 1979; Crossland et al., 2011). There is evidence that the neural processing of visual input from this preferred retinal location is enhanced by daily use. For example, JMD patients performed visual search better when target stimuli fell near their PRL. They also showed a task- and location-dependent upregulation of neural responses in early visual cortex (Plank et al., 2013). Visual stimulation of the PRL – in comparison to an area in the opposite hemifield (OppPRL) – with natural object pictures led to increased activation in brain regions responsible for object recognition (Plank et al., 2017). Also, Liu et al. (2010), who measured four JMD and four AMD patients under passive and active viewing conditions, found more extensive activation when stimulating the patients’ PRL in comparison to another retinal region with the same eccentricity. Stable eccentric fixation at the PRL appears to be an important moderating factor and prerequisite for high visual performance in visual search tasks (Plank et al., 2013) and for significant increases in activations in early and higher visual areas (Plank et al., 2017). Furthermore, the re-referencing of saccadic eye movements to the PRL (e.g., White and Bedell, 1990) has been associated with the successful use of this eccentric area for attentive encoding of objects into long-term memory (LTM; Geringswald et al., 2015), an ability usually ascribed to foveal inspection (e.g., Hollingworth, 2006). Normally sighted viewers with simulated central scotomas, who do not develop a “PRL” due to insufficient practice in extrafoveal scene exploration, had impaired performance in an LTM task (Geringswald et al., 2016). These observations may be the consequence of an intense, though mostly implicit, form of procedural (oculomotor) and perceptual learning, associated with the usage of a PRL. Crossland et al. (2005) found that all 25 MD patients in their experimental group formed a PRL within 6 months after disease onset. Sixteen of the patients (64%) were unaware of the adjustment that led to using that eccentric area of fixation. Nineteen of the patients used a consistent number of PRLs for all gaze positions, and eleven formed multiple fixation loci. However, reading speed appeared to be independent of the number and location of the PRLs. But patients, who were unaware of the coping strategy of the PRL and showed a consistent number of PRLs in all gaze positions, showed better reading speed. All in all, little is known about the formation of PRLs in people with central vision loss. According to a study by Farzaneh et al. (2021), who used a Nidek MP-1 microperimeter and Image J software to evaluate the fixation characteristics plus optical coherence tomography to determine the location of the central fovea in patients with AMD, the participants most frequently placed their PRL in the inferior and left visual field, which would result in a scotoma displacement to the superior and right visual field. Fixation stability was statistically similar in different locations of PRL, but improved with decreasing distance between PRL and fovea. Investigations in normally sighted people with simulated scotomas showed that especially individually different positions in the visual field with high attentional capabilities formed a PRL at this connected brain area (Barraza-Bernal et al., 2017). For example, participants with high attentional capabilities in the upper hemifield developed PRLs in the upper hemifield, those with high attentional capabilities in the lower hemifield developed PRLs in the lower hemifield and so on. The authors also showed, that trainings to use a PRL for fixating objects while a central scotoma is simulated – e.g., as here in eight 10-min blocks – show a PRL-forming effect.

For adapting to the new viewing conditions under central vision loss, we assume that the cortical eye fields are of importance. The cortical eye fields are primarily responsible for eye movements and shifts of attention (Grosbras and Paus, 2002). Since both brain hemispheres have eye fields, a distinction is made between right and left eye fields. The primary eye fields are referred to as the frontal eye fields and are located in BA 6 of the frontal lobe around the lateral part of the precentral sulcus (Kimmig et al., 2001; Leigh and Zee, 2006). It receives its input from the posterior visual areas, inferior parietal cortex, superior colliculi, thalamus, dorsolateral prefrontal cortex, and other eye fields. In turn, it projects to the contralateral FEF and to the supplemental and parietal eye fields (Leigh and Zee, 2006). Its main tasks include controlling ballistic gaze shifts (saccades) by initiating or inhibiting them and controlling attention. If lesions occur in the frontal eye fields, the execution of voluntary saccades is primarily affected, for example in the form of prolonged latency (Leigh and Zee, 2006). In addition, there is a functional link between the left FEF and short-term memory for target locations (Campana et al., 2007; Raabe et al., 2013), as well as a link with visuo-motor integration, requiring for visually guided movements (Wolynski et al., 2009). Research in non-human primates further showed that the FEF may be sub-divided into at least two parts (Tian and Lynch, 1996; Petit and Haxby, 1999; Krauzlis, 2004; De Castro et al., 2021): A medial part likely specialized in controlling smooth eye movements (FEFsem) and a lateral part likely specialized in saccadic eye movements (FEFsac). In close exchange with the FEFs are the supplementary eye fields (SEFs). Also located in the frontal lobe, they lie on the dorsomedial surface of both hemispheres. In addition to exchanging information with the FEFs, the SEFs are innervated by other cortices and the thalamus and also transmit back to these same cortices, as well as to the superior colliculi and the nucleus caudatus (Huerta and Kaas, 1990). The SEFs are involved in the planning and execution of saccades (Stuphorn et al., 2000). In particular, the SEFs play a key role in the control of eye movements when multiple competing saccade responses are possible, but not when routine saccade sequencing is performed (Parton et al., 2007). Another eye field has been suggested in the inferior part of the precentral sulcus, ventral to the FEF, the premotor eye field (PEF; e.g., Amiez and Petrides, 2009; Savaki et al., 2015; Schall et al., 2020). Coiner et al. (2019) refer to this region also as the inferior FEF (iFEF) in their review on the human eye movement network. The premotor eye field (PEF) was also identified in multi-modal parcellations of the human cortex (Glasser et al., 2016) and has been found in intraoperative stimulation during awake surgery to induce transient saccadic eye movements, together with, among others, the FEF (Pallud et al., 2018). It has been proposed to be the human homolog to the monkey premotor eye field (e.g., Petit and Pouget, 2019; Schall et al., 2020) and as such has been implicated in the representation of visual-oculomotor space, that is controls the direction of saccades and visual targets spatially (Savaki et al., 2015). According to Coiner et al. (2019), the precise function of PEF (or iFEF, as it is called in their review) remains elusive. Thus, the PEF has been implicated in functions jointly with the FEF, like pursuit eye movements and volitional saccades (e.g., Berman et al., 1999) as well as visually guided eye and head movements (Petit and Beauchamp, 2003). Additionally, the PEF can be activated during eye blinks (Kato and Miyauchi, 2003). Since macular degeneration causes blindness in the central visual field, which is responsible for stable fixation and visual acuity, examining the impact of the disease on the eye fields, which are responsible for saccades and attention shifts, is important to understand the changes of visual processing and visual performance in patients with central vision loss.

In this study, we were interested in possible structural alterations, specifically in CT alterations as a consequence of central vision loss. To this end, we calculated CT measures in representation areas of the PRL and an equally eccentric area in the opposite hemifield (OppPRL) in early visual cortex V1 and V2 in a group of AMD and JMD patients as well as an age-matched group of normally sighted controls. The PRL and OppPRL representation areas were determined by fMRI in each individual patient. We hypothesized that the CT at the PRL representation area may exceed the CT measured at the OppPRL area due to the special role the PRL plays in the patients’ daily vision. Additionally, we were interested in CT measures of the eye fields and how they correspond to behavioral adaptive measures like reading speed and fixation stability at the PRL of the patients. This hypothesis is motivated by an earlier finding of our group (Plank et al., 2011), where a whole brain regression analysis with behavioral measures as regressors revealed a significant correlation between fixation stability and gray matter volume in a cluster in superior and middle frontal gyri of the right hemisphere. As this research question was posed in a *post hoc* analysis, regions of interest for the frontal (FEF), supplementary (SEF), and premotor (PEF) eye fields have been determined functionally in an independent sample of 40 subjects, who performed a localizer task (block design: saccades vs. fixation) while measured with fMRI. We hypothesized to find positive correlations between behavioral measures, like reading speed and fixation stability, and CT measures in the eye fields of the patient group.

## Materials and Methods

### Participants

The data of 32 patients (P) with central scotomas due to hereditary retinal dystrophies or age-related macular degeneration (18 males, 14 females; mean age 53.4 years, range 19–84 years; see Table 1 for details) were included in this study. They are a subgroup with established PRLs of the sample included in the analysis of Beer et al. (2020). Their data were compared to a group of carefully age-matched normally sighted controls (C) (19 males, 13 females; mean age 52.2 years, range 23–83 years; see also Table 1 for details). There was no significant difference in age between the patient and control group [*t*(62) = 0.26; *p* = 0.79; *d* = 0.07]. All participants signed an informed consent form prior to the study and received monetary compensation for their participation. The study was approved by the Ethical Committee of the University of Regensburg and conducted in accordance to the ethical guidelines of the Declaration of Helsinki.

**TABLE 1:** Characteristics of patients (P1–P32) and controls (C1–C32) according to age, gender, and (for the patient group only) duration of disease in years, diagnosis, study eye, scotoma size (rounded diameter in degrees visual angle), decimal visual acuity, reading speed (in words per minute), and fixation stability (percentage of fixation in 2 and 4 degrees visual angle around fixation target).

Participant no.	Subgroup	Age	Gender	Disease duration (years)	Diagnosis	Study eye	Scotoma size (diameter in degrees visual angle)		Decimal visual acuity	Reading speed (wpm)	Fixation stability	
							Isopter III/4e	Isopter I/4e			2°	4°
P1	JMD	19	f	9	Stargardt	OS	15	30	0.05	110	0	1.90
P2	JMD	24	f	11	Stargardt	OS	20	20	0.05	132	86	100
P3	JMD	25	f	8	Stargardt	OD	20	20	0.08	77	20	57
P4	JMD	25	m	8	Stargardt	OD	10	25	0.1	98	100	100
P5	JMD	29	m	5	Stargardt	OD	10	10	0.1	76	95	100
P6	JMD	33	m	8	Cone-rod D	OS	25	25	0.08	27	100	100
P7	JMD	35	f	6	Stargardt	OD	10	10	0.1	83	98	100
P8	JMD	41	f	28	Cone-rod D	OS	25	25	0.1	19	83	100
P9	JMD	43	m	24	Stargardt	OS	20	20	0.1	60	67.09	75.27
P10	JMD	43	f	9	Stargardt	OD	15	15	0.1	78	77.64	92.61
P11	JMD	43	f	28	Stargardt	OD	10	20	0.1	60	25.64	27
P12	JMD	44	f	29	CACD	OD	10	25	0.05	57	14.4	36
P13	JMD	45	m	23	Stargardt	OS	10	10	0.2	96	96	100
P14	JMD	50	m	18	Cone D	OD	10	10	0.1	56	39.33	66.67
P15	JMD	53	m	23	MD	OD	10	20	0.08	137	18.35	21.88
P16	JMD	55	m	16	Stargardt	OD	15	15	0.1	83	30.92	57.67
P17	JMD	59	m	13	Cone-rod D	OS	10	10	0.1	58.67	90	100
P18	JMD	59	m	16	Cone-rod D	OD	10	10	0.1	80	19.43	20.48
P19	JMD	65	m	6	Cone-rod D	OS	30	35	0.05	14	78	97
P20	JMD	65	m	17	Cone-rod D	OS	30	30	0.1	31	43.5	48
P21	JMD	66	m	13	Stargardt	OD	30	40	0.05	22	6.42	8.33
P22	AMD	55	m	3	AMD	OD	10	20	0.1	15.33	71	97
P23	AMD	62	m	5	AMD	OS	20	20	0.1	63	43	98
P24	AMD	63	m	6	AMD	OS	10	10	0.1	34.67	16	43
P25	AMD	63	f	8	AMD	OD	20	20	0.1	51.67	73	94
P26	AMD	70	f	2	AMD	OD	15	15	0.06	38.33	51	88
P27	AMD	72	f	12	AMD	OS	10	10	0.1	38.33	33	72
P28	AMD	78	m	4	AMD	OS	10	10	0.1	81.67	54	86
P29	AMD	79	m	8	AMD	OD	15	15	0.2	41.67	28	84
P30	AMD	80	f	15	AMD	OS	10	10	0.2	37.67	85	96
P31	AMD	81	f	21	AMD	OS	10	20	0.2	15.67	94	99
P32	AMD	84	f	6	AMD	OS	15	15	0.1	31.33	18	42
C1	CTL_JMD	23	f	–	–	–	–		–	–	–	–
C2	CTL_JMD	23	m	–	–	–	–		–	–	–	–
C3	CTL_JMD	23	f	–	–	–	–		–	–	–	–
C4	CTL_JMD	26	m	–	–	–	–		–	–	–	–
C5	CTL_JMD	28	m	–	–	–	–		–	–	–	–
C6	CTL_JMD	35	m	–	–	–	–		–	–	–	–
C7	CTL_JMD	35	m	–	–	–	–		–	–	–	–
C8	CTL_JMD	37	m	–	–	–	–		–	–	–	–
C9	CTL_JMD	38	m	–	–	–	–		–	–	–	–
C10	CTL_JMD	40	m	–	–	–	–		–	–	–	–
C11	CTL_JMD	43	m	–	–	–	–		–	–	–	–
C12	CTL_JMD	45	m	–	–	–	–		–	–	–	–
C13	CTL_JMD	51	f	–	–	–	–		–	–	–	–
C14	CTL_JMD	52	f	–	–	–	–		–	–	–	–
C15	CTL_JMD	54	f	–	–	–	–		–	–	–	–
C16	CTL_JMD	55	f	–	–	–	–		–	–	–	–
C17	CTL_JMD	59	m	–	–	–	–		–	–	–	–
C18	CTL_JMD	60	m	–	–	–	–		–	–	–	–
C19	CTL_JMD	62	f	–	–	–	–		–	–	–	–
C20	CTL_JMD	63	m	–	–	–	–		–	–	–	–
C21	CTL_JMD	68	f	–	–	–	–		–	–	–	–
C22	CTL_AMD	54	f	–	–	–	–		–	–	–	–
C23	CTL_AMD	56	f	–	–	–	–		–	–	–	–
C24	CTL_AMD	62	f	–	–	–	–		–	–	–	–
C25	CTL_AMD	63	f	–	–	–	–		–	–	–	–
C26	CTL_AMD	64	f	–	–	–	–		–	–	–	–
C27	CTL_AMD	70	m	–	–	–	–		–	–	–	–
C28	CTL_AMD	71	m	–	–	–	–		–	–	–	–
C29	CTL_AMD	71	m	–	–	–	–		–	–	–	–
C30	CTL_AMD	78	m	–	–	–	–		–	–	–	–
C31	CTL_AMD	78	m	–	–	–	–		–	–	–	–
C32	CTL_AMD	83	m	–	–	–	–		–	–	–	–

*m, male; f, female; Stargardt, Stargardt’s disease; CACD, central areolar choroidal dystrophy; MD, unclassified hereditary macular dystrophy; Cone D, cone dystrophy; Cone-rod D, cone-rod dystrophy; OS, oculus sinister; OD, oculus dexter; wpm, words per minute.*

### Clinical Characteristics and Visual Field Measurements of MD Patients

Table 1 presents a detailed description of patients and controls, including the diagnosis, duration of disease, visual acuity, scotoma size, reading speed and fixation stability. All characteristics were measured as described previously (Plank et al., 2011, 2013, 2017). Best-corrected visual acuity was determined by using a Vision Screener (Rodenstock Rodavist 524/S1) and Eye Charts for distant visual acuity (Oculus Nr. 4616) and near visual acuity (Zeiss/Frohnhäuser). One eye was chosen for stimulation during fMRI and other measurements (study eye). The dominant eye was preferably chosen for the study eye. In patients P1 and P4 the non-dominant eye was chosen, because it was the better eye and/or the one with higher fixation stability.

**FIGURE 1:**
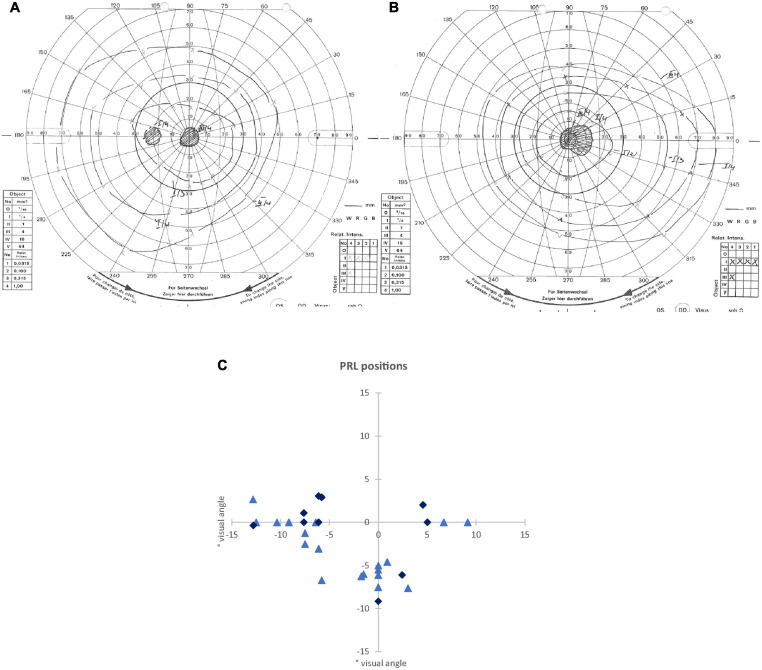
**(A,B)** Examples of visual field measurements using Goldmann perimetry: **(A)** for the left eye of an AMD patient (P24), where scotoma borders of isopters III/4e and I/4e were identical. **(B)** For the right eye of a JMD patient (P11), where different scotoma borders resulted from isopters III/4e and I/4e. In this patient, the blind spot could not be detected independently of the scotoma from isopter I/4e. **(C)** Schematic depiction of the positions of the preferred retinal loci (PRLs) in visual field of each of the 32 patients (some PRL positions overlapping with each other). The PRL positions of the JMD patients are marked with light blue triangles, the PRL positions of the AMD patients are marked with dark blue diamonds.

The scotoma size was measured using kinetic Goldmann perimetry with the isopters III/4e, I/4e, I/3e, I/2e, and I/1e. The reliability of the Goldmann perimetric measures depends on fixation stability. We defined two measures for scotoma sizes. As edges of the scotomata, those points were marked, where isopter III/4e or isopter I/4e were no longer detected, respectively. The two scotoma sizes are reported in Table 1 as scotoma diameter in degrees of visual angle as an average and approximation of rounded vertical and horizontal dimensions. In nine patients, scotoma sizes determined by use of isopters III/4e and I/4e differed. Typical examples for Goldmann perimetry are given in Figure 1.

To measure fixation stability, we used a Nidek MP-1 microperimeter (Nidek Co, Japan). Patients were requested to fixate a red cross of 4 degrees visual angle in diameter with their preferred eccentric location on the retina (PRL) for on average 30 s. The technique measures 25 samples per second, so that 750 samples of fixation points result over a time period of 30 s. During the measurement the camera sometimes lost track of the subject’s eye. This can be due to eye blinks or fixation instability in the form of large saccades. The Nidek software records the time period that was measured and the proportion of the time span that was effectively tracked, as well as the percentages of fixation points that fell in a range of 2° or 4° diameter visual angle around the center of the target, based on the time spans effectively tracked. Thus, fixation stability can be overestimated by long or frequently interrupted time spans where the camera lost track of eye position due to large saccades. To compensate for this, we corrected the given fixation stability as described in Plank et al. (2011). These corrected values are given in Table 1.

We determined the position of PRLs according to the resultant Nidek images. This was later verified using a video eyetracker (High Speed Video Eyetracker Toolbox, Cambridge Research Systems, United Kingdom), while the participants fixated a visual target on a computer monitor. Fourteen patients had a PRL located in the left visual field, 13 patients used a PRL in the lower visual field, and four patients a PRL in the right visual field. One patient (P 13) used two different PRLs deliberately for certain tasks, one in the lower visual field and one in the left visual field. For the analysis here, we only considered the PRL in the left visual field of patient 13 that seemed more appropriate for the functional localizer used here, containing stimulation with flickering checkerboards and object pictures. Figure 1 presents an overview of the PRL positions of all patients.

To estimate reading speed, patients read aloud a continuous text for 3 min (as described in Plank et al., 2011), which was recorded. We then counted the number of words read and calculated the mean of words read per minute. These values are given in Table 1. All participants read the same text, taken from a book [German translation of Lessing (2003): The Grandmothers], printed on a sheet of paper (font: Arial, font size: 10 pt, single spaced). Patients used magnification glasses customized to their needs.

### Magnetic Resonance Imaging Data Acquisition

All magnetic resonance imaging (MRI) data were acquired by a 3 Tesla Siemens Allegra head scanner (Siemens AG, Erlangen, Germany) over a period of 2 years. For each participant one high-resolution T1-weighted structural image was acquired. Additionally, regions of interest (ROIs) of the PRL and OppPRL areas in visual cortex were identified by functional MRI. The anatomical T1-weighted images (repetition time: 2250 ms, echo time: 2.6 ms, flip angle: 9°, voxel size: 1 × 1 × 1 mm^3^, field of view: 256 × 256 mm^2^) were acquired by an MPRAGE (magnetization prepared rapid acquisition gradient echo) sequence across 160 sagittal slices. Functional MRI was performed by a T2^∗^-weighted gradient-echo sequence with echoplanar read-out (repetition time: 2000 ms, echo time: 30 ms, flip angle: 90° voxel size: 3 × 3 × 3 mm^3^, field of view: 192 × 192 mm^2^). The 34 axial slices covered most of the brain including the entire occipital cortex.

### Cortical Reconstruction

T1-weighted structural images were automatically reconstructed by Freesurfer version 5.3 (Martinos Center for Biomedical Imaging, Charlestown, MA, United States; Fischl, 2012). The reconstruction followed procedures as previously described (Beer et al., 2020). In brief, T1-weighted images were intensity normalized and automatically segmented into gray and white matter structures. Then, the boundary between white and gray matter (WGB) was automatically tessellated and corrected for topologic inaccuracies. Finally, the cortical surface was deformed, inflated, and registered to a spherical atlas preserving the individual folding patterns of sulci and gyri. Moreover, the reconstruction provides several macroscopic brain measures including CT. For our analysis, CT was calculated for defined ROIs.

### Regions of Interest Analysis

**FIGURE 2:**
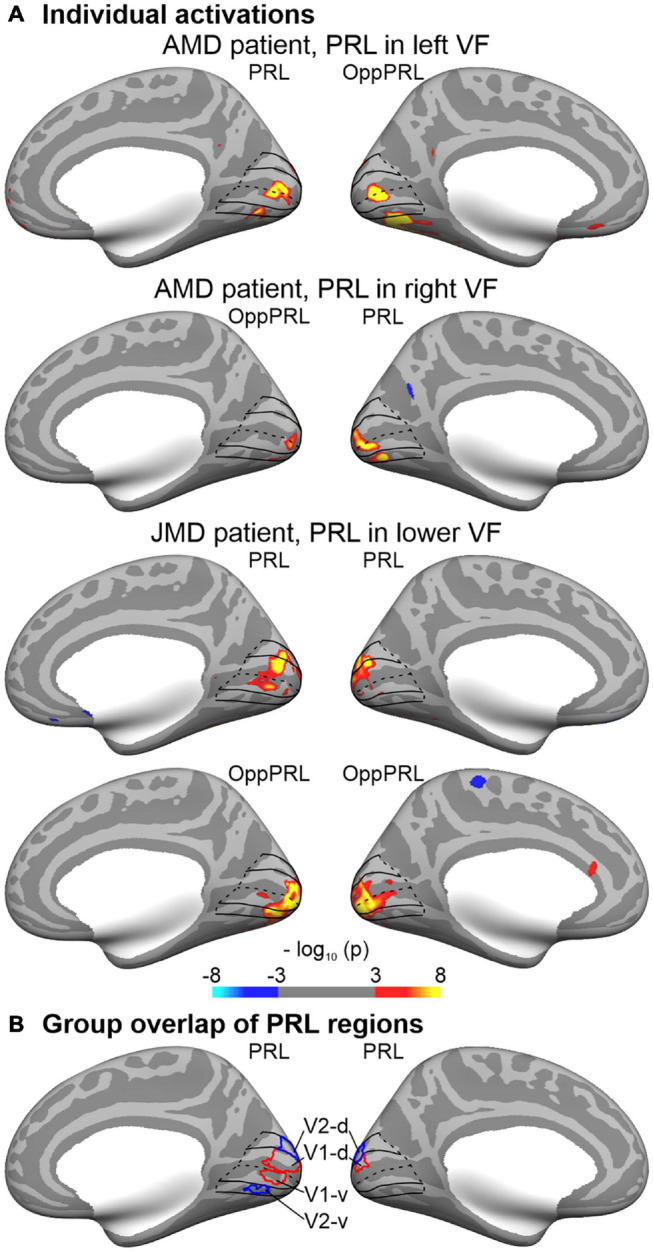
Regions-of-interest (ROIs) of the preferred retinal location (PRL). **(A)** Individual functional MRI maps show significant activation for the stimulation of the PRL part of the visual field or the control region (OppPRL), respectively, for three representative patients with a PRL in the left, right, or lower visual field (VF), respectively. All maps were thresholded (*p* ≤ 0.001; uncorrected) and color-coded (red/yellow: stimulation > baseline; blue: stimulation < baseline). In order to facilitate comparison, individual maps were projected (by spherical registration) to the inflated cortical surface of the Freesurfer average brain. Only the most relevant views (medial, hemispheres contralateral to stimulation) are depicted. ROIs of the PRL and OppPRL regions were identified in each patient based on the individual maps (see text for details) and further sub-divided into V1 and V2 parts based on a retinotopic template (Benson et al., 2014). The outlines of V1 and V2 are shown in black lines for reference. Note that dorsal (d) and ventral (v) parts were combined for the cortical thickness analyses. **(B)** The outlines show the group overlap of at least 8 (out of 32) selected PRL regions in V1 (red) and V2 (blue).

The cortical representation of the PRL was estimated in each individual brain by functional MRI while stimulating the PRL with flickering checkerboard stimuli as described in Plank et al. (2017). Moreover, a control ROI (OppPRL) was identified by stimulating visual field locations opposite to the PRL (mirrored either at the vertical or horizontal meridian, respectively). For 16 patients the OppPRL was mirrored at the vertical meridian, for nine patients the OppPRL was mirrored at the horizontal meridian, and for seven patients the OppPRL was mirrored at both, vertical and horizontal meridian. As described in more detail in Plank et al. (2017), in the paradigm used, we stimulated the PRL, the OppPRL and the central area in the visual field with flickering checkerboards and object pictures. The participants had no explicit task in this paradigm apart from viewing the stimulation. Stimuli were radial black and white checkerboards (size: 9° × 9° visual angle) presented with a flicker rate of 8 Hz or static chromatic images of natural objects (e.g., tools, vehicles, animals, musical instruments) (size: 7.3° × 7.3° visual angle). For determining the ROIs for PRL and OppPRL representation areas the activation elicited by the flickering checkerboard stimuli was used. Stimuli were presented blockwise on a gray background, together with a baseline condition (gray background of mean luminance). The blocks were presented in four cycles, flickering checkerboards and object pictures were presented in blocks of 13 s each, the baseline condition in blocks of 18 s. The patients conducted all paradigms monocularly with their study eye while the other eye was patched. They had to direct their fovea to the center of the screen. Because the patients had central scotomas we presented auxiliary stimuli to ensure fixation. Depending on how well they could consciously perceive their scotoma and/or how well they were accustomed to fixate with their PRL, these auxiliary stimuli were adapted to the individual needs of the patients. The auxiliary stimuli consisted of four red dots (each about 0.7° visual angle in diameter) positioned at the edges of the respective scotoma at eccentricities that were visible to each individual patient and/or the fixation target (letter “X”) at the position of the PRL, adapted in color and size to the needs of the patients. In the same session (see Plank et al., 2017), also eccentricity mapping and meridian mapping was conducted with flickering checkerboard stimuli, which allowed the definition of the individual representation areas of V1d/v, V2d/v, and V3d/v. Representation areas for PRL and OppPRL were obtained by contrasting the checkerboard stimulation of PRL and OppPRL against the baseline condition (mean luminance). To this end, significance maps were overlaid on the individual reconstructed surface together with the individually determined borders of V1d/v, V2d/v, and V3d/v. Borders of the PRL and OppPRL ROIs were always drawn excluding the central ROI. The evoked activation [thresholded at *p* < 0.001 (uncorrected), but for some patients a reduced threshold of *p* < 0.01 had to be adopted] determined the eccentricity and extent of PRL and OppPRL representation areas on the individual retinotopic grid in which ROIs with contiguous voxels were drawn throughout those individually determined portions of V1, V2, and V3. For the analysis conducted here, PRL and OppPRL ROIs were pooled over those V1, V2, and V3 portions and then again sub-divided into V1 and V2 areas based on a retinotopic atlas derived from functional MRI (Benson et al., 2014). Dorsal and ventral representations were pooled for left and right PRLs, left and right representations were pooled for upper and lower visual field PRLs. PRL and OppPRL labels as determined for each individual patient were mapped onto the cortical surface of the Freesurfer average brain (fsaverage) by spherical registration (Fischl et al., 1999) and from there mapped onto the cortical surface of each patient’s age-matched control subject. This standardized, atlas-based approach appeared to us to be more appropriate for the CT analysis conducted here. Thus, CT measures of the respective ROIs in visual cortex representing PRL (V1: average number of vertices = 857.34, *SD* = 396.48; V2: average number of vertices = 685.37, *SD* = 307.42) and OppPRL (V1: average number of vertices = 1038.62, *SD* = 472.55; V2: average number of vertices = 802.84, *SD* = 323.08) positions could be compared between patients and controls. Average number of vertices in PRL and OppPRL ROIs did not differ significantly from each other [V1: *t*(31) = 1.95; *p* = 0.060; *d* = 0.34; V2: *t*(31) = 1.89; *p* = 0.067; *d* = 0.33]. Figure 2A shows individual functional MRI maps with significant activation for the stimulation of the PRL and OppPRL region for three representative patients with a PRL in the left, right, or lower visual field, respectively. Figure 2B shows the outlines of a group overlap of PRL ROIs in V1 and V2. Borders of V1 and V2 including their subdivisions (ventral and dorsal) were determined by use of the retinotopic atlas (Benson et al., 2014) and are shown for reference. The outlines show the PRL overlap of at least eight (out of 32) patients. Thus, it indicates the approximate locations of the most typical PRL ROIs (right hemisphere or dorsal parts). Due to the variation across individual ROIs, the overlapping area (V1: 1294 vertices, V2: 1020 vertices) exceeds the size of the average ROIs. Note that for patients with their PRLs located at the horizontal meridian dorsal and ventral parts of V1 and V2 were combined in the ROIs.

**FIGURE 3:**
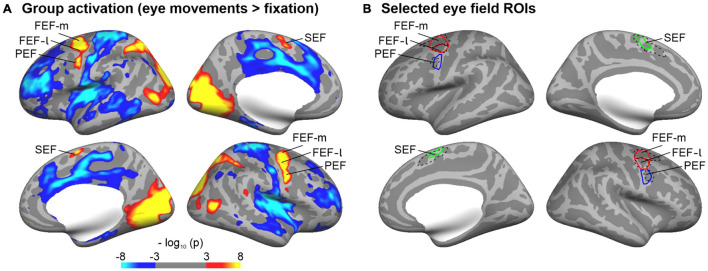
Regions-of-interest (ROIs) of cortical representations of the eye fields. **(A)** Eye fields were identified in an independent sample that performed an eye movement task during functional MRI. The group maps show brain regions with significant (thresholded at *p* ≤ 0.001; FDR corrected) signal differences for the comparison eye movement versus fixation blocks (red/yellow: eye movements > fixation; blue: eye movements < fixation) overlaid on the inflated cortical surface of the Freesurfer average brain. **(B)** Selected ROIs of the eye fields (FEF-m, FEF-l, PEF, SEF). Eye fields were identified on the group maps thresholded at *p* ≤ 0.001 (see **A**). In order to delineate the boundaries between FEF and PEF, an enhanced threshold (*p* ≤ 0.0001) was adopted. The FEF was further sub-divided into a medial and lateral part as it overlapped with two separate parcellations (6a, FEF) of a multimodal imaging atlas (Glasser et al., 2016). The relevant atlas parcellations are shown in black dashed lines for reference.

The cortical representations of the eye fields (frontal eye fields, FEF; supplementary eye fields, SEF; premotor eye fields, PEF) of each hemisphere were identified by functional MRI in an independent sample (*N* = 40, college-aged students). Participants of that sample had to direct their gaze to a small dot, which jumped randomly to one of six different locations on the horizontal axis of the screen (see also Frank et al., 2014). This task was shown to reliably activate the eye fields (Kimmig et al., 2001). Blocks of saccades alternated with blocks of central fixation. There were overall 48 blocks with a duration of 12 s each (24 blocks of saccades and fixation, respectively). ROIs for the eye fields were identified on the cortical surface of the Freesurfer average brain based on a random-effects surface-based group analysis. Statistical parametric maps for the contrast saccades > fixation were mapped to the cortical surface (thresholded at *p* < 0.001, FDR-corrected). This contrast showed reliable activation patterns in the lateral and medial frontal cortex, which were delineated and labeled as FEF, PEF, and SEF, respectively. Research in non-human primates showed that the FEF may be sub-divided into at least two parts (Tian and Lynch, 1996; Petit and Haxby, 1999; Krauzlis, 2004; De Castro et al., 2021). Although this distinction still needs to be verified in humans, our functionally defined FEF overlapped with two distinct cortical areas of a multimodal cortical atlas (Glasser et al., 2016). Accordingly, we sub-divided our FEF into a medial (FEF-m) and lateral (FEF-l) part. Figure 3 shows the labels of the eye fields obtained from this functional analysis on the Freesurfer average brain.

## Results

### Behavioral Results

Table 2 presents the correlation results (Pearson correlation coefficients and *p*-values) of demographic and behavioral data in the patient group.

**TABLE 2:** Pearson correlation coefficients (r) together with their respective *p*-values (italic values, *p*) of demographic and behavioral measures (two-sided), for the whole group of patients (*N* = 32) and for the subgroups (AMD, *N* = 11; JMD, *N* = 21), respectively.

			*Duration of disease*	*Scotoma size Isopter III/4e*	*Scotoma size Isopter I/4e*	*Visual acuity*	*Reading speed*	*Fixation stability (2*°*)*	*Fixation stability (4*°*)*
**All patients (*N* = 32)**									
	***Age***	r	–0.103	–0.032	–0.182	**0.408**	–**0.553**	–0.187	0.005
		*p*	*0.576*	*0.862*	*0.319*	***0.021***	***0.001***	*0.305*	*0.977*
	***Duration of disease***	r		–0.059	0.116	0.174	0.070	–0.103	–0.288
		*p*		*0.747*	*0.526*	*0.340*	*0.705*	*0.575*	*0.109*
	***Scotoma size (III/4e)***	r			**0.748**	–**0.366**	–0.341	–0.015	–0.019
		*p*			**<*0.001***	***0.039***	*0.056*	*0.937*	*0.918*
	***Scotoma size (I/4e)***	r				–**0.462**	–0.214	–0.163	–0.287
		*p*				***0.008***	*0.240*	*0.372*	*0.111*
	***Visual acuity***	r					–0.162	0.315	**0.362**
		*p*					*0.376*	*0.079*	***0.042***
	***Reading speed***	r						–0.047	–0.156
		*p*						*0.799*	*0.393*
	***Fixation stability (2*°*)***	r							**0.882**
		*p*							**<*0.001***
**AMD (*N* = 11)**									
	***Age***	r	0.492	–0.229	–0.372	0.535	0.032	–0.008	–0.180
		*p*	*0.125*	*0.499*	*0.260*	*0.090*	*0.925*	*0.981*	*0.597*
	***Duration of disease***	r		–0.330	<0.001	**0.759**	–0.377	0.505	0.219
		*p*		*0.322*	*1.00*	***0.007***	*0.254*	*0.113*	*0.518*
	***Scotoma size (III/4e)***	r			0.553	–0.258	0.351	–0.144	0.153
		*p*			*0.078*	*0.443*	*0.290*	*0.672*	*0.654*
	***Scotoma size (I/4e)***	r				<0.001	–0.271	0.389	0.486
		*p*				*1.00*	*0.421*	*0.236*	*0.130*
	***Visual acuity***	r					–0.273	0.392	0.296
		*p*					*0.417*	*0.233*	*0.376*
	***Reading speed***	r						–0.187	0.105
		*p*						*0.582*	*0.758*
	***Fixation stability (2*°*)***	r							**0.800**
		*p*							***0.003***
**JMD (*N* = 21)**									
	***Age***	r	0.282	0.277	0.143	0.062	–**0.481**	–0.237	–0.237
		*p*	*0.216*	*0.224*	*0.535*	*0.791*	***0.027***	*0.301*	*0.301*
	***Duration of disease***	r		–0.152	–0.025	0.249	–0.082	–0.325	–0.314
		*p*		*0.509*	*0.913*	*0.277*	*0.725*	*0.151*	*0.166*
	***Scotoma size (III/4e)***	r			**0.754**	–0.380	–**0.621**	–0.012	0.016
		*p*			**<*0.001***	*0.089*	***0.003***	*0.959*	*0.945*
	***Scotoma size (I/4e)***	r				–**0.609**	–0.420	–0.310	–0.342
		*p*				***0.003***	*0.058*	*0.171*	*0.129*
	***Visual acuity***	r					0.113	0.400	0.357
		*p*					*0.626*	*0.072*	*0.112*
	***Reading speed***	r						–0.067	–0.101
		*p*						*0.774*	*0.663*
	***Fixation stability (2*°*)***	r							**0.955**
		*p*							**<*0.001***

*Significant results are shown in bold font. No correction for multiple comparison was applied to the *p*-values.*

Overall, reading speed was negatively correlated with age and with scotoma size, but only in the JMD group. Fixation stability was positively correlated with visual acuity. We also tested for group differences between the JMD and AMD patient group for the demographic and behavioral measures by use of independent sample *t*-Tests. The JMD patients were on average, as expected, significantly younger [*t*(30 = –5.7; *p* < 0.001; *d* = 1.5] than the AMD patients, but had on average a significantly longer disease duration [*t*(30) = 2.6; *p* = 0.015; *d* = 0.88] and a significantly better reading speed [*t*(29.8) = 2.9; *p* = 0.006; *d* = 0.86]. The two patient groups did not differ significantly in visual acuity, scotoma size and fixation stability (all *p* > 0.05).

### Magnetic Resonance Imaging Results

#### Cortical Thickness Measures in Preferred Retinal Locus (PRL) and OppPRL Regions

**FIGURE 4:**
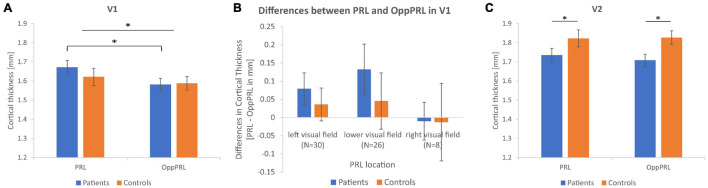
Mean cortical thickness measures in mm together with their respective standard errors for the PRL and OppPRL regions in V1 **(A)** and V2 **(C)**. In V1, the repeated-measures ANOVA yielded a significant main effect of ROI, with greater CT values in PRL ROIs, that was more pronounced in the patient group, as *post hoc* tests revealed. In V2, the repeated-measures ANOVA yielded a significant main effect of group, with greater CT values in the control group independently from ROI. **(B)** Shows the mean differences in CT between PRL and OppPRL ROIs plotted separately for the three PRL location clusters in the left, lower and right visual field (see also Figure 1C) together with their respective standard errors (^∗^*p* < 0.05).

Figure 4 shows the mean CT measures at the PRL and the OppPRL regions in V1 and V2 for the patient and control group.

First, we tested for overall effects with a repeated-measures ANOVA for the within-subjects factors visual cortex (V1, V2) and ROI (PRL, OppPRL) and the between-subjects factor group (patients, controls). We obtained a significant main effect of visual cortex [*F*(1,62) = 49.9; *p* < 0.001; η^2^ = 0.45], with overall higher CT in V2 compared to V1, and a significant interaction between visual cortex and group [*F*(1,62) = 7.7; *p* = 0.007; η^2^ = 0.11] with the control group exhibiting higher CT values in V2, though the effect size of 0.11 suggests a small effect here. The main effects of ROI [*F*(1,62) = 2.7; *p* = 0.107; η^2^ = 0.01] and group [*F*(1,62) = 0.96; *p* = 0.331; η^2^ = 0.01] were not significant. Also the interactions visual cortex × ROI [*F*(1,62) = 1.6; *p* = 0.208; η^2^ = 0.02], ROI × group [*F*(1,62) = 0.99; *p* = 0.324; η^2^ = 0.02], and visual cortex × ROI × group [*F*(1,62) = 0.10; *p* = 0.757; η^2^ = 0.002] were not significant. Subsequently, we conducted the ANOVAs separately for primary (V1) and secondary (V2) visual cortex.

In V1, the repeated-measures ANOVA with the within-subjects factor ROI (PRL, OppPRL) and the between-subjects factor group (patients, controls) revealed a significant main effect of ROI [*F*(1,62) = 5.4; *p* = 0.024; η^2^ = 0.080] with overall higher CT at PRL in comparison to OppPRL, but the effect size again points to a small effect. The main effect of group [*F*(1,62) = 0.23; *p* = 0.634; η^2^ = 0.004] and the interaction between ROI and group [*F*(1,62) = 1.1; *p* = 0.289; η^2^ = 0.018] were not significant. *Post hoc* paired-sample *t*-Tests between PRL and OppPRL separately for each group revealed that the main effect ROI was mainly driven as expected by the patient group [*t*(31) = 2.5; *p* = 0.017; *d* = 0.44; control group: *t*(31) = 0.85; *p* = 0.404; *d* = 0.15]. After splitting the patient group into its subgroups JMD and AMD, paired-sample *t*-Tests in V1 yielded a significant effect in the JMD group [*t*(20) = 2.1; *p* = 0.049; *d* = 0.46] but not in the AMD group [*t*(10) = 1.4; *p* = 0.197; *d* = 0.42] between the two ROIs.

To further clarify the effect in V1 between PRL and OppPRL, we examined possible location effects independently of disease. To this end, we divided the participants in clusters according to PRL location (left visual field, *N* = 30; lower visual field, *N* = 26; right visual field, *N* = 8), pooled over patients and controls (see Figure 1C). If a PRL fell into a quadrant instead of falling directly on a meridian, group classification depended on the meridian with the larger eccentricity. We conducted a repeated-measures ANOVA with the within-subjects factor ROI (PRL, OppPRL) and the between-subjects factor PRL location (left, lower, right), which yielded neither a significant main effect of ROI [*F*(1,61) = 2.0; *p* = 0.164; η^2^ = 0.031], nor a significant main effect of PRL location [*F*(2,61) = 2.0; *p* = 0.143; η^2^ = 0.023], nor a significant interaction ROI × PRL location [*F*(2,61) = 0.72; *p* = 0.490; η^2^ = 0.023]. Also *post hoc* paired comparisons between the three PRL locations (Bonferroni corrected) revealed no significant differences (all *p* > 0.05). Figure 4B shows that positive differences between PRL and OppPRL CT values are most pronounced in the subgroups with the PRL in the left and lower visual field, while the subgroup with the PRL in the right visual field is characterized by a small sample size (*N* = 8) and high variance in CT values. For that reason, in an additional analysis we excluded the subgroup with the PRL in the right visual field and found a significant main effect of ROI [*F*(1,54) = 6.2; *p* = 0.016; η^2^ = 0.104] and a significant main effect of PRL location [*F*(1,54) = 4.2; *p* = 0.046; η^2^ = 0.072], where the group with the PRL in the lower visual field exhibited overall greater CT values, albeit with small effect sizes. The interaction between ROI and PRL location was not significant [*F*(1,54) = 0.29; *p* = 0.592; η^2^ = 0.005].

In V2, the repeated-measures ANOVA with the within-subjects factor ROI (PRL, OppPRL) and the between-subjects factor group (patients, controls) revealed no significant main effect of ROI [*F*(1,62) = 0.13; *p* = 0.720; η^2^ = 0.002], but a significant main effect of group [*F*(1,62) = 544; *p* = 0.039; η^2^ = 0.067], with higher CT values in the control group, though the effect size again suggests a small effect. The interaction ROI × group was not significant [*F*(1,62) = 0.25; *p* = 0.622; η^2^ = 0.004]. *Post hoc* paired-sample *t*-Tests showed that CT in V2 between PRL and OppPRL ROIs differed neither in the patient group [*t*(31) = 0.72; *p* = 0.476; *d* = 0.13], nor in the control group [*t*(31) = –0.08; *p* = 0.933; *d* = 0.01]. Additionally, in V2, both patient groups showed no significant effect between PRL and OppPRL [JMD: *t*(20) = 0.20; *p* = 0.845; *d* = 0.04; AMD: *t*(10) = 0.84; *p* = 0.418; *d* = 0.25].

#### Cortical Thickness Measures in Eye Fields

**FIGURE 5:**
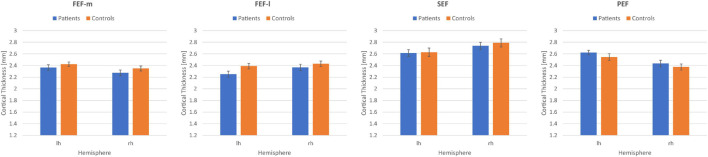
Mean cortical thickness measures in mm together with their respective standard errors for the eye fields FEFm, FEFl, SEF, and PEF in the left (lh) and right (rh) hemisphere for the patient group (*N* = 32) and the control group (*N* = 32).

We determined the overall effects for the within-subjects factors eye fields (FEF-m, FEF-l, SEF, PEF) and hemisphere (lh, rh; repeated-measures ANOVA) and the between-subjects factor group (patients, controls). We obtained a significant main effect of eye fields [*F*(3,186) = 60.2; *p* < 0.001; η^2^ = 0.49], a significant interaction eye fields × hemisphere [*F*(2.3,143.7) = 14.6; *p* < 0.001; η^2^ = 0.19; Greenhouse–Geisser corrected], and a significant interaction eye fields × group [*F*(3,186) = 3.2; *p* = 0.025; η^2^ = 0.05]. The main effects hemisphere [*F*(1,62) = 0.26; *p* = 0.61; η^2^ = 0.004] and group [*F*(1,62) = 0.35; *p* = 0.558; η^2^ = 0.01], as well as the interactions hemisphere × group [*F*(1,62) = 0.003; *p* = 0.957; η^2^ < 0.001] and eye fields × hemisphere × group [*F*(2.3,143.7) = 0.44; *p* = 0.673; η^2^ = 0.01; Greenhouse–Geisser corrected] were not significant. Figure 5 shows the mean CT measures for the eye fields FEF-m, FEF-l, SEF and PEF in both hemispheres for the patient group and the control group. *Post hoc t*-Tests for each eye field ROI between patients and controls revealed no significant group differences (all *p* > 0.05).

Subsequently, as an explorative analysis, we tested for significant correlations between demographic and behavioral data of the patient groups and CT measures in the eye fields FEF-m, FEF-l, SEF, and PEF of both hemispheres. Pearson correlations were done two-sided for demographic variables age, duration of disease, scotoma size and visual acuity, and were done one-sided for behavioral variables reading speed and fixation stability, because we had a directional (one-tailed) hypothesis for the latter three. Since we assumed compensational structural processes in the eye fields, we expected to find positive correlations between those behavioral measures and CT. Table 3 gives the resultant Pearson correlation coefficients together with their respective *p*-values (in italic font; no correction for multiple comparisons was applied).

**TABLE 3:** Pearson correlation coefficients together with their respective *p*-values (italic numbers) of behavioral data and cortical thickness in medial and dorsal parts of the frontal eye fields (FEF-m and FEF-l), supplementary eye fields (SEF), and premotor eye fields (PEF) of the left and right hemisphere, respectively.

ROI	Hemisphere	Group		Age (two-sided)	Duration of disease (two-sided)	Scotoma size (two-sided) Isopter III/4e	Scotoma size (two-sided) Isopter I/4e	Visual acuity (two-sided)	Reading speed (wpm) (one-sided)	Fixation stability (2°) (one-sided)	Fixation stability (4°) (one-sided)
**FEF-m**	**lh**	***All***	r	**–0.505**	0.100	0.129	0.202	–0.138	**0.303**	0.157	0.081
			*p*	***0.003***	*0.587*	*0.483*	*0.268*	*0.452*	***0.046***	*0.196*	*0.329*
		***AMD***	r	–0.532	–0.066	0.278	0.311	0.009	–0.009	0.097	0.502
			*p*	*0.092*	*0.846*	*0.407*	*0.353*	*0.978*	*0.490*	*0.389*	*0.058*
		***JMD***	r	–0.277	–0.122	–0.072	< 0.001	0.044	0.286	0.190	0.072
			*p*	*0.224*	*0.598*	*0.757*	*0.999*	*0.850*	*0.105*	*0.205*	*0.378*
	**rh**	***All***	r	**–0.648**	0.118	0.126	0.205	–0.263	**0.356**	**0.317**	0.176
			*p*	**< *0.001***	*0.520*	*0.492*	*0.261*	*0.145*	***0.023***	***0.038***	*0.168*
		***AMD***	r	–0.425	0.020	0.275	0.337	–0.152	–0.003	0.391	**0.527**
			*p*	*0.192*	*0.954*	*0.412*	*0.311*	*0.655*	*0.497*	*0.117*	***0.048***
		***JMD***	r	**–0.569**	–0.141	–0.050	0.003	–0.072	0.280	0.296	0.267
			*p*	***0.007***	*0.543*	*0.830*	*0.990*	*0.757*	*0.109*	*0.096*	*0.121*
**FEF-l**	**lh**	***All***	r	**–0.613**	–0.003	0.212	0.217	–0.322	0.291	0.223	0.166
			*p*	**< *0.001***	*0.987*	*0.243*	*0.233*	*0.072*	*0.053*	*0.110*	*0.182*
		***AMD***	r	**–0.729**	–0.361	0.326	0.531	–0.362	–0.083	0.084	0.480
			*p*	***0.011***	*0.275*	*0.327*	*0.093*	*0.274*	*0.404*	*0.403*	*0.068*
		***JMD***	r	**–0.435**	–0.166	0.070	–0.055	0.012	0.262	0.306	0.252
			*p*	***0.049***	*0.472*	*0.762*	*0.813*	*0.958*	*0.125*	*0.088*	*0.135*
	**rh**	***All***	r	**–0.681**	0.206	0.213	0.339	–0.290	0.174	0.188	0.085
			*p*	**< *0.001***	*0.259*	*0.241*	*0.058*	*0.107*	*0.170*	*0.151*	*0.323*
		***AMD***	r	**–0.761**	–0.151	0.260	0.400	–0.167	–0.071	0.327	0.518
			*p*	***0.007***	*0.659*	*0.440*	*0.223*	*0.624*	*0.418*	*0.163*	*0.051*
		***JMD***	r	**–0.533**	0.098	0.089	0.203	–0.111	–0.002	0.105	0.113
			*p*	***0.013***	*0.672*	*703*	*0.378*	*0.633*	*0.496*	*0.325*	*0.312*
**SEF**	**lh**	***All***	r	**–0.502**	0.140	0.146	0.286	–0.169	0.229	–0.013	–0.060
			*p*	***0.003***	*0.445*	*0.425*	*0.112*	*0.354*	*0.103*	*0.472*	*0.371*
		***AMD***	r	–0.545	–0.016	0.111	0.335	–0.082	–0.063	–0.170	0.132
			*p*	*0.083*	*0.963*	*0.746*	*0.314*	*0.810*	*0.427*	*0.309*	*0.350*
		***JMD***	r	–0.250	–0.061	0.043	0.150	0.064	0.128	0.010	–0.0003
			*p*	*0.275*	*0.793*	*0.854*	*0.517*	*0.784*	*0.290*	*0.482*	*0.499*
	**rh**	***All***	r	**–0.434**	–0.021	0.343	**0.407**	–0.114	0.133	**0.351**	**0.298**
			*p*	***0.013***	*0.910*	*0.054*	***0.021***	*0.536*	*0.234*	***0.025***	***0.049***
		***AMD***	r	–0.356	–0.011	0.326	0.540	0.302	–0.171	0.368	**0.595**
			*p*	*0.282*	*0.973*	*0.328*	*0.086*	*0.367*	*0.308*	*0.132*	***0.027***
		***JMD***	r	–0.338	–0.255	0.313	0.318	–0.291	0.073	0.350	0.350
			*p*	*0.133*	*0.264*	*0.167*	*0.160*	*0.201*	*0.377*	*0.060*	*0.060*
**PEF**	**lh**	***All***	r	–0.272	0.211	–0.160	0.015	–0.209	0.221	–0.118	–0.203
			*p*	*0.133*	*0.246*	*0.383*	*0.937*	*0.252*	*0.112*	*0.261*	*0.133*
		***AMD***	r	–0.443	–0.538	0.396	0.085	–0.579	–0.175	–0.421	–0.371
			*p*	*0.172*	*0.088*	*0.228*	*0.804*	*0.062*	*0.303*	*0.099*	*0.130*
		***JMD***	r	–0.179	0.425	–0.371	–0.066	0.147	0.285	0.459	–0.125
			*p*	*0.437*	*0.055*	*0.097*	*0.775*	*0.525*	*0.106*	*0.459*	*0.295*
	**rh**	***All***	r	**–0.449**	–0.273	0.025	0.198	–0.346	0.273	–0.016	–0.126
			*p*	***0.010***	*0.130*	*0.891*	*0.279*	*0.052*	*0.066*	*0.465*	*0.247*
		***AMD***	r	**–0.618**	–0.277	0.312	0.416	–0.397	–0.433	–0.079	–0.213
			*p*	***0.043***	*0.410*	*0.351*	*0.203*	*0.226*	*0.092*	*0.408*	*0.264*
		***JMD***	r	**–0.531**	–0.386	–0.084	0.121	–0.296	**0.462**	–0.004	–0.077
			*p*	***0.013***	*0.084*	*0.718*	*0.600*	*0.193*	***0.018***	*0.494*	*0.370*

*Significant results are shown in bold font. No correction for multiple comparison was applied to the *p*-values. Sample sizes were All: N = 32; AMD: N = 11; JMD: N = 21.*

Specifically, we found positive correlations between reading speed and CT in the left and right FEF-m and the right PEF, that was more pronounced in the JMD group, and between fixation stability and CT in the right FEF-m and the right SEF. Since reading speed was negatively correlated with age and with scotoma size (for isopter III/4e) in the JMD group (see Table 2), the positive correlation with CT in left and right FEF-m and right PEF may at least partially be confounded with those variables. To clarify this issue, we conducted an additional partial correlation between reading speed and CT values in FEF-m and PEF with the control variables age and scotoma size (III/4e). In fact, that partial correlation yielded a non-significant result for the FEF-m ROIs for the whole group of patients [left: *r* = 0.100; *p* = 0.300; right: *r* = 0.063, *p* = 0.370], whereas for the PEF ROI the correlation with reading speed in the JMD group was still visible [*r* = 0.397; *p* = 0.046]. Fixation stability, on the other hand, was significantly correlated with visual acuity (see Table 2). When we controlled for visual acuity in a partial correlation, the significant results regarding positive correlations between fixation stability and CT in the eye fields FEF-m and SEF, became even more pronounced [FEF-m rh: fixation stability within 2°: *r* = 0.437, *p* = 0.007; fixation stability within 4°: *r* = 0.301, *p* = 0.050; SEF rh: fixation stability within 2°: *r* = 0.410, *p* = 0.011; fixation stability within 4°: *r* = 0.366; *p* = 0.021]. The analyses concerning fixation stability were all conducted with the whole group of patients (*N* = 32). Since a large portion of our patient group are JMD patients, and among them the largest group are Stargardt patients (*N* = 11), who differ from the other diseases in their etiology (e.g., Glazer and Dryja, 2002), we recalculated the correlations for the Stargardt group (*N* = 11) alone. Thereby we found an additional positive correlation between reading speed and CT in right SEF [*r* = 0.622; *p* = 0.015], that still holds in a partial correlation with age as covariate [*r* = 0.568; *p* = 0.034] and is not visible in the other patient sub-groups.

## Discussion

In this study we investigated differences in CT in relation to the preferred retinal locus (PRL) in early visual cortex (V1, V2, Figure 4), and in selected ROIs involved in the control of eye movements in the left and right hemisphere (Figure 5). As an exploratory analysis, we also correlated reading speed and eccentric fixation stability in patients with central vision loss with CT in oculomotor ROIs (Table 3). The results point to differences in early visual cortex, where the PRL projection zone exhibits greater CT compared to a region opposite of this location, but it is unclear, to what extent these differences depend on natural differences in CT between those regions already existing pre-disease, or indeed on neuroplastic changes as a consequence of disease. We also found a trend to positive correlations between CT in oculomotor ROIs and reading speed and fixation stability in patients with central vision loss.

### Cortical Thickness of the Preferred Retinal Locus Representation Area

The PRLs’ representation areas and – for comparison – an equally eccentric area in the opposite hemifield (OppPRL) were determined with functional MRI by employing local stimulation with flickering checkerboards (see Plank et al., 2017), individually for each patient. Those cortical coordinates were then mapped to the brains of age-matched normally sighted controls, on a patient-by-patient basis. The analysis revealed that indeed, on average, the PRL representation area in V1 in patients exhibited greater CT values than the OppPRL representation area (see Figure 4A). This effect was more pronounced in the JMD group, who had, on average, longer disease durations, and was limited to V1. In V2, this pattern of results was not observed (see Figure 4C). Although the *post hoc* tests revealed no significant differences between PRL and OppPRL CT values in the control group, where CT was measured in comparable ROIs, there was also a trend to positive differences in CT between PRL and OppPRL ROIs in controls, which resulted in a significant main effect of ROI in primary visual cortex (V1). Since the interaction between ROI and group was not significant, we additionally tested for PRL location, independently of group. To this end, we divided our sample according to the clusters visible in Figure 1C in the PRL locations left, lower and right visual field, pooled over patients and controls. The analyses conducted here indeed revealed a significant effect of ROI (CT at PRL > CT at OppPRL) for the PRL locations in left and lower visual field. Figure 4B also illustrates that result by plotting the mean differences between PRL and OppPRL for the patient and control group as a function of PRL location. Our assumption was, that CT especially at the representation area of the PRL of patients should be enhanced as a consequence of the compensatory use of this specific area of intact peripheral retina as a pseudo fovea in daily visual tasks. Though, as Figure 4B shows, the difference in CT between PRL and OppPRL was more pronounced in the patient group, no significant differences to the control group were observed. As such it is unclear, whether the greater CT values at PRL locations in the patient group reflect compensatory, neuroplastic changes, or whether most patients intuitively chose PRL locations that were supported by premorbid greater CT in the respective retinotopic areas of V1. While it seems likely that these effects interacted with each other, further research is warranted to clarify the causality of these effects. In functional findings, we and others could already observe enhanced processing when the PRL was stimulated in visual discrimination and search tasks (e.g., Liu et al., 2010; Plank et al., 2013, 2017). And several studies have also shown that structural changes in gray matter can occur as a consequence of perceptual learning or other forms of training (e.g., Draganski et al., 2004; Ceccarelli et al., 2009; Engvig et al., 2010; Schmidt-Wilcke et al., 2010; Krafnick et al., 2011; Wenger et al., 2012; Ditye et al., 2013; Kühn et al., 2014). As such, CT enhancements at the representation area of the PRL in V1 could be interpreted as a consequence of a kind of perceptual learning process at this location in the visual field due to its persistent use for daily visual tasks over the years. On the other hand, there is, of course, no further evidence for any causal relationship. As we did not do a longitudinal analysis, we cannot be certain, whether the greater CT at the PRL representation area could be a consequence of enhanced use or whether a greater CT (pre-disease) at that specific location in V1 possibly favored the establishment of a patient’s PRL just at that corresponding retinal locus. A trend to a comparable effect in the control group points to that possibility. And on the other hand, studies have already shown, that greater CT in a relevant brain area pre-learning could favor the subsequent learning of related tasks. For example, Frank et al. (2016) reported that preexisting individual differences in CT in motion-sensitive area V5 and in posterior parietal cortex that were involved in a perceptual learning task could predict task-specific learning rates. Bi et al. (2014) reported a similar result regarding CT in the left fusiform face area and learning rates in a facial view discrimination task. As such, it might well be the case that CT in earlier visual areas like V1 could promote processing of visual stimuli stemming from the respective retinotopic area in the visual field and that patients with central vision loss intuitively choose that particular retinal area as their preferred pseudo fovea (PRL). Further research would be needed to clarify these two possible effects. For example, in a longitudinal study with MD patients starting early in their disease before the establishment of a distinct PRL CT in visual cortex could be measured, and the results could be probed in regard to the further development of the patients’ eccentric viewing strategies.

Another factor leading to differences between PRL and OppPRL CT in the patient group could be related to degenerative processes at the OppPRL representation area rather than compensatory effects at the PRL representation area. Degenerative processes at the OppPRL representation area could stem from less intact retina due to e.g., asymmetric scotomas or irregular scotoma borders or from less usage of the OppPRL area in daily vision despite intact retina. As outlined above, the PRL as a kind of pseudo fovea is usually characterized by stronger visual abilities than other intact retinal areas, but to exclude that merely degenerative processes at the OppPRL site due to less intact retina drive the observed effect, it should be controlled for overall usable vision at the OppPRL area. The fMRI paradigm used to determine the ROIs (Plank et al., 2017) was a passive viewing paradigm, where the patients did not perform a visual task, so that we could not derive performance levels based on these measurements. On the other hand, from almost all of our patients we also had results from a visual search task (*N* = 30), where they were requested to detect a target letter L among distractor letters T, as well as from a single letter control task (*N* = 29), where on each trial the letter L or T had to be discriminated at any of 16 positions in the peripheral visual field. Both tasks are described in detail in Plank et al. (2013). From these tasks, hit rates from targets in the PRL area (six letter positions near the patient’s PRL) and from targets in the OppPRL area (six letter positions near the patient’s OppPRL) could be derived. The remaining two patients did not perform that visual search tasks, but took part in a perceptual learning experiment (Plank et al., 2014), where hit rates in a texture discrimination task could be derived from the first session at the PRL position and the OppPRL position of the two patients. As such we can compare hit rates from at least one visual task performed at both locations from each patient. Since the PRL is a well-trained area that patients use for daily vision, overall better performance at the PRL could be expected. If the OppPRL has usable vision, which points to overall intact retina, the discrepancy between hit rates at both locations should not be too large. Therefore, we calculated the difference in hit rates between PRL and OppPRL in all patients and estimated the CT comparison between PRL and OppPRL ROIs in V1 again while we excluded all patients where the difference in hit rate on at least one of the visual tasks was greater than 0.40. A difference in hit rates of greater than 0.40 between PRL and OppPRL in at least one of the tasks could be observed in eight patients (P8, P9, P16, P19, P22, P24, P26, P31). After exclusion of these eight patients, the analysis on differences in CT between PRL and OppPRL in V1 in the remaining 24 patients remained significant (*p* = 0.02; *d* = 0.51). Thus, we consider it less likely that those differences in CT are due to any retinal degenerative processes at the OppPRL area cutting off input to the respective visual cortex. Nevertheless, reduced usage of the OppPRL area in comparison to the PRL area in daily vision and associated differences in vision could still underlie these findings. Our analyses cannot differentiate between those two possible effects. These observations are retrospective to disease onset, so any statement concerning causality would require further evidence from a prospective study of the effects of central vision loss on CT.

There were no overall significant differences in CT between patients and controls in our analyses in V1. As such, we did not replicate the result by Burge et al. (2016), who reported that patients with central vision loss showed significantly greater CT values in peripheral areas of V1 compared to that exhibited by the control group. On the other hand, we only investigated one specific eccentricity in every patient and his/her respective control, corresponding to the projection zone of the individually determined PRL, so our analysis does not provide information about CT measures at other eccentricities in our participants. We did observe a significant overall difference in CT between patients and controls in V2, with the controls exhibiting thicker cortex than the patients, but the effect size was rather small, and it was also limited to the specific eccentricities examined here.

### Cortical Thickness of the Cortical Eye Fields

A previous study of our group (Plank et al., 2011) found a significant correlation between fixation stability and gray matter volume in a cluster of the right superior and middle frontal gyri. The current study investigated CT and its correlations with behavioral data in the frontal (FEF), supplementary (SEF) and premotor eye fields (PEF). In order to clarify which of these regions are most relevant, we adopted a region-of-interest approach. ROIs of these eye fields were defined by fMRI activity elicited by an eye movement task in an independent sample. We compared these ROIs with an atlas of cortical parcellations based on multimodal MRI (Glasser et al., 2016). Because our functionally defined FEF in the superior and middle frontal gyri overlapped with two distinct parcellations, we further subdivided our FEF into a medial (FEF-m overlapping with area 6a in the atlas parcellation) and a lateral part (FEF-l overlapping with area FEF in the atlas parcellation), respectively.

The mean CT of these eye fields did not significantly differ between our patient and control group. This argues both against a systematic enhancement or degeneration as a result of central vision loss in the cortical structure of the eye fields. In most of the eye fields considered here, CT inversely correlated with age (see Table 3), a known and often replicated phenomenon all over the cortex (e.g., Salat et al., 2004; Lemaitre et al., 2012; Beer et al., 2020). We were especially interested in correlations between compensatory behavioral measures like reading speed and eccentric fixation stability in the patients and CT in the eye fields, for which we expected a positive correlation. Indeed, we found positive correlations between reading speed and CT in the left and right FEF-m and the right PEF, both driven by data from the JMD group (see Table 3). Reading speed also correlated negatively with age and scotoma size, and an additional partial correlation with the control variables age and scotoma size revealed that the correlation in the left and right FEF-m may be confounded by those variables. On the other hand, the correlation in the right PEF remained at least borderline significant (*r* = 0.397; *p* = 0.046) even when age and scotoma size were controlled for. As such, this finding could be a sign of an at least partial relationship between reading speed and CT in the right PEF, but caution is warranted in interpreting these effects, since those *p*-values were not adjusted for multiple comparisons. The PEF is implicated in visually guided eye and head movements (Petit and Beauchamp, 2003), among other functions, which are usually important for normal reading and which have to be adapted, when a central scotoma impairs reading. This hints to a possible role of the right PEF in achieving good reading speed despite a central scotoma. But further research is needed in this regard. Additionally, we found positive correlations between fixation stability within 2° visual angle around fixation target and CT in the right FEF-m and the right SEF for the entire patient group. The AMD patients, who had on average a shorter disease duration, exhibited a similar result for fixation stability within 4° visual angle around fixation target and CT in right FEF-m and right SEF (see Table 3). Since fixation stability was positively correlated with visual acuity, we additionally conducted a partial correlation with visual acuity as a control variable. In that analysis, the correlations were reconfirmed and strengthened, but again caution is warranted in interpreting the effects, because the *p*-values were not adjusted for multiple comparisons. According to Coiner et al. (2019), the frontal eye fields are implicated in various eye movements, especially in the planning and initiation of saccades and in maintaining fixation. Research in non-human primates suggest a subdivision of FEF into a saccade-related and a pursuit-related part (Tian and Lynch, 1996; Petit and Haxby, 1999; De Castro et al., 2021; Krauzlis, 2004). Accordingly, we subdivided our FEF into a medial (FEF-m) and a lateral (FEF-l) part. Interestingly, we found the most pronounced correlation between fixation stability and CT in the medial subregion (FEF-m) of the right hemisphere. The medial part (FEF-m) also corresponds better to the ROI, we reported earlier in JMD patients, in which fixation stability correlated with gray matter volume (Plank et al., 2011). The JMD subject samples of these two studies largely overlapped (*N* = 20). In the current analysis, however, also the AMD group, that was not involved in the former study, showed a correlation between fixation stability and CT in that ROI (see Table 3). Positive correlations between fixation stability and CT were also obtained for the supplementary eye fields (SEF) of the right hemisphere, though the significance would again not survive correction for multiple comparisons. According to Coiner et al. (2019), the SEF are also implicated in the preparation and execution of saccadic and pursuit eye movements, but they appear to be involved in more cognitively demanding tasks, like predictive eye movements (e.g., Heide et al., 2001; Alvarez et al., 2010), performing saccade sequences (Heide et al., 2001; Thakkar et al., 2014) or combinations of saccades and body movements (Petit and Beauchamp, 2003). Additionally, we found a trend for a positive correlation between CT in the right SEF and scotoma size (Table 3), that may also be a sign for compensatory adjustment. Thus, the right FEF-m and SEF might work together in guiding eye movement behavior in patients with central vision loss, especially with regard to maintaining fixation at the eccentric PRL instead of the fovea. Connolly et al. (2000) found a similar cluster, a bit anterior to FEF, using fMRI that was especially involved in anti-saccades. Lepsien and Pollmann (2002) found a cluster in the right SEF and FEF active during covert reorienting and inhibition of return in a spatial cueing paradigm. The authors discussed this activation in the light of the hypothesis that inhibition of return is caused by inhibitory oculomotor processes (Taylor and Klein, 1998). Moreover, Milea et al. (2007) linked activation in the dorsolateral prefrontal cortex and the right presupplementary eye field and frontal eye fields to inhibitory processes in saccadic eye movements. Maintaining fixation at the eccentric PRL may involve inhibition of making eye movements to the originally favored fovea, thus leading to a thicker cortex in the eye fields as a supporting factor. Additionally, these regions (among others) have been implicated in oculomotor learning (Grosbras et al., 2001). Further research should tackle the role of re-referencing of saccadic eye movements to the PRL instead of the fovea (e.g., White and Bedell, 1990) in neuroplasticity of the eye fields. In our patients, we did not explicitly test, if a complete shift of the oculomotor reference from the fovea to the PRL had occurred in all or just a part of them. Without a shift of the oculomotor reference to the PRL, patients would for example re-center gaze to the fovea and then correct this position to the PRL in saccadic tasks (see also e.g., White and Bedell, 1990). Future experiments could reveal, if CT measures might relate directly to an adaptation process leading to re-referencing to the PRL. As stated above, we only found correlations between CT in the eye fields and behavioral data, which as such do not provide any information with respect to a causal relationship. The trend to a thicker cortex associated with greater reading speeds and fixation stability could reflect oculomotor learning in the adaptation process leading to consistent eccentric viewing. Note that our experimental design does not allow us to rule out the notion that patients, who had a pre-existing thicker cortex in the eye fields, could benefit from it in such an adaptation process.

## Conclusion

In this study, we investigated how extensive eccentric viewing associated with central vision loss and the development of a PRL on intact peripheral retina, functioning as a pseudo fovea, affects brain structures responsible for visual perception and visually guided eye movements. Previous studies had shown that central vision loss can result in a degeneration of cortical gray matter at the occipital pole representing the central visual field (e.g., Boucard et al., 2009; Plank et al., 2011; Hernowo et al., 2014; Prins et al., 2016b; Beer et al., 2020). On the other hand, CT of early visual cortex turned out to be largely preserved or even enhanced in representational areas of the peripheral visual field (Burge et al., 2016). Here we could show that the representation area of the PRL in V1 exhibited significantly greater CT than a control area in the opposite hemifield (OppPRL). This effect was more pronounced in the patient group, but also the age-matched controls contributed to it. Thus, further research is needed to clarify, if increased CT at PRL representation areas in V1 is a consequence or a prerequisite for successful adaptation of that retinal area to compensate for central vision loss. Additionally, we found a trend toward positive correlations between CT in the right FEF and SEF and fixation stability and in the right PEF and reading speed. These results point to an association between the efficiency of compensatory strategies used by patients with central scotomas and structural properties of the brain, notably in early visual cortex and cortical areas underlying the control of eye movements. These findings may have implications for rehabilitation measures and possibly point to the neuroplastic capacities of the brain to adapt to the demands of eccentric viewing. However, further research employing longitudinal designs would be needed to confirm a causal link between CT alterations and such behavioral adaptive processes.

## Data Availability Statement

The raw data supporting the conclusions of this article will be made available by the authors, without undue reservation.

## Ethics Statement

The studies involving human participants were reviewed and approved by Ethikkommission bei der Universität Regensburg, Universität Regensburg, 93040 Regensburg. The patients/participants provided their written informed consent to participate in this study.

## Author Contributions

TP and MG designed the study, supervised the data acquisition and analysis, drafted and edited the manuscript and figures, and acquired third-party funding. EB, AB, SB, MM, SF, and HJ contributed to different aspects of patient recruitment, patient diagnosis and clinical examination, data acquisition, analysis and visualization. All the authors commented on final version of the manuscript.

## Conflict of Interest

The authors declare that the research was conducted in the absence of any commercial or financial relationships that could be construed as a potential conflict of interest.

## Publisher’s Note

All claims expressed in this article are solely those of the authors and do not necessarily represent those of their affiliated organizations, or those of the publisher, the editors and the reviewers. Any product that may be evaluated in this article, or claim that may be made by its manufacturer, is not guaranteed or endorsed by the publisher.
